# Nitric Oxide Sensing by a Blue Fluorescent Protein

**DOI:** 10.3390/antiox11112229

**Published:** 2022-11-11

**Authors:** Chiara Montali, Stefania Abbruzzetti, Arne Franzen, Giorgia Casini, Stefano Bruno, Pietro Delcanale, Sandra Burgstaller, Jeta Ramadani-Muja, Roland Malli, Thomas Gensch, Cristiano Viappiani

**Affiliations:** 1 Dipartimento di Scienze Matematiche, Fisiche e Informatiche, Università di Parma, Parco Area delle Scienze 7A, 43124 Parma, Italy; 2 Institute of Biological Information Processing (IBI-1: Molecular and Cellular Physiology), Forschungszentrum Jülich, Leo-Brandt-Straße, D-52428 Jülich, Germany; 3 Dipartimento di Scienze degli Alimenti e del Farmaco, Università di Parma, Parco Area delle Scienze 23/A, 43124 Parma, Italy; 4 Molecular Biology and Biochemistry, Gottfried Schatz Research Center, Medical University of Graz, Neue Stiftingtalstraße 6/6, 8010 Graz, Austria

**Keywords:** fluorescent protein, nitric oxide, cysteine, S-nitrosylation, fluorescence lifetime, fluorescence quenching, biosensor

## Abstract

S-Nitrosylation of cysteine residues is an important molecular mechanism for dynamic, post-translational regulation of several proteins, providing a ubiquitous redox regulation. Cys residues are present in several fluorescent proteins (FP), including members of the family of *Aequorea victoria* Green Fluorescent Protein (GFP)-derived FPs, where two highly conserved cysteine residues contribute to a favorable environment for the autocatalytic chromophore formation reaction. The effect of nitric oxide on the fluorescence properties of FPs has not been investigated thus far, despite the tremendous role FPs have played for 25 years as tools in cell biology. We have examined the response to nitric oxide of fluorescence emission by the blue-emitting fluorescent protein mTagBFP2. To our surprise, upon exposure to micromolar concentrations of nitric oxide, we observed a roughly 30% reduction in fluorescence quantum yield and lifetime. Recovery of fluorescence emission is observed after treatment with Na-dithionite. Experiments on related fluorescent proteins from different families show similar nitric oxide sensitivity of their fluorescence. We correlate the effect with S-nitrosylation of Cys residues. Mutation of Cys residues in mTagBFP2 removes its nitric oxide sensitivity. Similarly, fluorescent proteins devoid of Cys residues are insensitive to nitric oxide. We finally show that mTagBFP2 can sense exogenously generated nitric oxide when expressed in a living mammalian cell. We propose mTagBFP2 as the starting point for a new class of genetically encoded nitric oxide sensors based on fluorescence lifetime imaging.

## 1. Introduction

Cysteine (Cys), one of the least abundant amino acids, is usually highly conserved within the functional sites of proteins. Cys residues play a crucial role in the stabilization of protein structures, particularly through the formation of intramolecular disulfide bridges and through the coordination of metal centers in cofactors such as the heme [1]. Moreover, Cys residues were suggested to tune thermal stability and to have a role in redox regulation [2,3]. For instance, tetrameric hemoglobins (Hb) in vertebrates have several Cys residues [4] that confer detoxifying properties, such as protection against reactive oxygen (ROS) and nitrogen (RNS) species, perhaps also shielding against electrophilic attack [4]. In several cases, these reactions impact the functional properties of proteins, with an important regulatory action through their remarkable sensitivity to the redox potential of the environment [5].

**Scheme 1:**
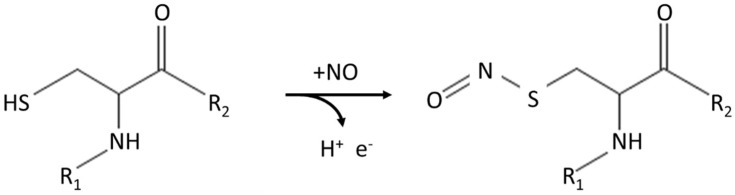
S-Nitrosylation of cysteine. Adapted from [11].

Among other modifications, S-nitrosylation of Cys residues in the presence of nitric oxide and oxygen (Scheme 1) is a commonly encountered reaction, with consequences on protein structure and dynamics in a variety of cases [6,7,8]. A well-known example is S-nitrosylation at Cys-β93 in human Hb [9]. It was proposed that NO acts as an ancestral regulator of diverse biological functions, acting throughout the phylogenetic tree [10]. In this sense, redox-active Cys residues may tune protein function by working as redox-sensing molecular switches that respond to ROS and RNS [11]. Thus, S-nitrosylation of cysteine residues may be regarded as an important molecular mechanism for dynamic, post-translational regulation of many proteins, providing a ubiquitous redox regulation [6,10].

Most fluorescent proteins (FPs) contain at least one cysteine residue. Members of the family of *Aequorea victoria* GFP-derived FPs contain two highly conserved cysteine residues located between the end of β-strand 3 and one end of the internal chromophore-containing α-helix. These two residues are deemed to contribute to the local environment required for the autocatalytic formation of the chromophore, which is responsible for the visible absorption and fluorescence properties of FPs [12]. Cys residues were also engineered to introduce sensitivity of the fluorescence properties to the redox potential of the protein environment [13,14,15,16]. Conversely, Cys-free fluorescent proteins were engineered for studies in oxidative environments [17].

Using mTagBFP2, a blue-emitting variant with improved emission and stability [18,19], as a prototype case, we demonstrated that fluorescence emission from this fluorescent protein (but also others; see below) exhibits a peculiar sensitivity to nitric oxide, which leads to fluorescence quenching with a small (around 30%) yet detectable and reproducible reduction in fluorescence intensity and lifetime. Reduction by Na-dithionite results in recovery of fluorescence emission. We correlated the effect with the presence of Cys residues near the protein chromophore since a mutation of Cys residues removes the NO sensitivity. Experiments on related fluorescent proteins from different families, devoid of Cys residues, demonstrated negligible sensitivity of their emission to NO. Other cysteine-containing GFP-like fluorescent proteins show similar NO- sensitivity of their fluorescence. We also prove that mTagBFP2 can sense NO when expressed in a living mammalian cell. This opens the way to the development of more sensitive mTagBFP2 and other FP variants that can be utilized to sense NO in physiologically relevant processes. We propose mTagBFP2 as genetically encoded NO sensors for fluorescence lifetime imaging applications. The small protein size and the dynamic range of its sensitivity represent important benefits over previously proposed genetically encoded fluorescent NO sensors [20,21]. Genetically encoded fluorescent sensors for monitoring intracellular NO levels have been recently reviewed [21]. The newly developed class of genetically encoded NO probes, named geNOps, consists of different FP variants directly fused to a bacterial NO-binding domain, referred to as the GAF domain. The GAF (cGMP-specific phosphodiesterases, adenylyl cyclases and FhlA) domain selectively binds NO via a non-heme iron(II) center. The basis of the NO sensitivity has been interpreted as follows. In geNOps, the NO-binding GAF domain brings the radical in close vicinity to the chromophore of the FPs, thus affecting the excited state and leading to a decrease in fluorescence emission. This process is fully reversible. In comparison to the FP proposed in this study, geNOps show higher signal dynamics and higher affinity for NO but has a more complex molecular structure. Another NO-sensitive, protein-based chimeric construct was obtained by fusing Cyan Fluorescent Protein (CFP) and Yellow Fluorescent Protein (YFP) to the C- or N-terminal ends of human type IIa metallothionein [22]. The binding of NO leads to a decreased FRET [22]. However, it has been observed that data on reversibility of this sensor are not conclusive. Nevertheless, geNOps have been used recently for insightful physiological studies [23]. Chimeric proteins comprising myoglobin as a sensing unit and either mCherry, or a tandem fusion between Enhanced Yellow Fluorescent Protein (EYFP) (or citrine) and mCherry, as fluorescent reporters were recently proposed as NO sensors. These constructs exploit changes in FRET between the fluorescent protein(s) and the heme upon metmyoglobin formation, as a consequence of the reaction of oxygenated ferrous myoglobin with NO [24,25,26]. The sensors proved suitable for FLIM imaging [27]. While these constructs afford a potentially useful NO probe for FLIM imaging, their larger size as well as the two or three protein domain structure (with two fluorescent proteins and the non-heme Fe-binding domain, respectively) add additional concerns and complexity to these sensors when compared to the simple fluorescent proteins (such as mTagBFP2) under investigation in this work.

## 2. Materials and Methods

### 2.1. Plasmid Construction

The construct pBAD-mTagBFP2 was provided by V.V. Verkusha (Albert Einstein College of Medicine, New York, NY, USA) via Addgene (Watertown, MA, USA; [#34632]).

The desired mutations of the single mutant pBADHisD-mTagBFP2 C26A and triple mutant pBADHisD-mTagBFP2 C26A C114S C222S were conducted by site-direct mutagenesis on the Addgene plasmid pBAD-mTagBFP2 [#34632], using an overlapping PCR strategy.

To obtain the prokaryotic expression construct pET11a-RFP-T-N, the coding region of pTagRFP-T-N (kind gift of Evrogen, Moscow, Russia) was amplified by PCR introducing a C-terminal 6xHis-Tag and was cloned into a pET11a vector (Merck, Darmstadt, Germany). The pET11a-pmCherry construct was generated in a similar same way using mTMEM16A-pmCherryN1 (kind gift of Björn Schröder, Max-Dellbrück-Center for Molecular Medicine, Berlin, Germany) as a template.

The final clones were sequenced commercially to confirm the mutations. The DNAs encoding mTagBFP2 and mTagBFP2 C26A C114S C222S, respectively, for expression and experiments in mammalian cells were subcloned from pBADHisD-vector into the FsY 1.1-eGFP G.W. vector (provided by Mikhail Filippov, Nizhny Novgorod, Russia).

The pcDNA3.1 C-geNOp vector [20] was used for expression of C-geNOp in HeLa cells.

### 2.2. Overexpression and Purification of Recombinant Proteins

Overexpression of recombinant proteins was performed for pBAD-mTagBFP2 and for its mutants encoding for the C26A and C26A, C114S, and C222S variants in the *E. coli* strain TOP10F (Invitrogen, Carlsbad, CA, USA). The starter cultures were grown in Luria–Bertani medium (LB medium) (Sigma, Deisenhofen, Germany) with antibiotics (ampicillin 100 µg/mL) (AppliChem, Darmstadt, Germany), grown at 37 °C overnight, and transferred into 500 mL LB with same antibiotics to reach an OD600 of ≈0.03. The bacteria were grown until they reached an optical density of 0.4–0.5 at 600 nm. At that point, protein expression was induced by the addition of arabinose (f.c. 0,02% *w*/*v*) (Sigma). Expression was further carried out at 37 °C for 4 h.

For the pET11a constructs (pET11a-RFP-T-N and pET11a-Cherry), the expression was performed in a similar way but with a different *E. coli* strain BL21 (DE3) Codon Plus RP (Stratagene California, La Jolla, CA, USA). The starter cultures were grown in LB medium with antibiotics (ampicillin 100 µg/mL, chloramphenicol 18 µg/mL; AppliChem), grown at 37 °C overnight, and then transferred into 500 mL LB to reach an optical density of ≈0.03 at 600 nm. The bacteria were grown until they reached an optical density of 0.4–0.5 at 600. At that point, protein expression was induced by the addition of isopropyl-D-thiogalactopyranoside (IPTG (AppliChem), 1 mM f.c.). Expression was further carried out over night at 20 °C (≈20 h).

The cells were harvested, processed, and purified according to the semi-batch purification protocol of polyhistidine-tagged proteins under native conditions using Protino^®^Ni-NTA agarose (Macherey-Nagel, Düren, Germany) or HisPur™ Ni-NTA Resin (ThermoFisher Scientific, Rodano (MI), Italy). Subsequently, the samples were concentrated, and simultaneously the buffer was exchanged for phosphate-buffered saline (PBS; pH 7.4) with a Centricon 10 (10 kDa Millipore Merck, Burlington, MA, USA) centrifugal filter unit. The integrity and function of the proteins were judged by Coomassie-blue-stained SDS-PAGE and spectrometric analysis.

### 2.3. Experiments

Protein solutions in phosphate-buffered saline (PBS), at pH 7.4, were used at the final concentration of around 1 µM.

MAHMA NONOate, 6-(2-hydroxy-1-methyl-2-nitrosohydrazino)-N-methyl-1-hexanamine (Sigma-Aldrich, Vienna, Austria) was used as a NO donor. For each measurement, the MAHMA NONOate powder was initially deoxygenated in a nitrogen flow in a gastight vial with PTFE/silicone septum and was subsequently dissolved in nitrogen-saturated PBS buffer. The quantity of powder and the volume of PBS were adjusted to have a NO final concentration of about 2 mM. After waiting for 20 min to allow complete decomposition of the NO donor, the concentrated NO solution was added to the protein solution using a Hamilton GASTIGHT^®^ syringe to reach the desired NO concentration.

To prevent oxygen leakage, the protein solution was kept in a gastight quartz cuvette with a reservoir and a cap with a septum.

Fluorescence excitation and emission spectra were collected with a Perkin-Elmer LS50 steady-state fluorimeter. Fluorescence decays were recorded with a FLS920 time-correlated single-photon counting (TCSPC) system (Edinburgh Instruments, Livingston, UK). Measurements were carried out using pulsed LED excitation at 380 nm for mTagBFP2 (PicoQuant PLS 380, 750 ps), 450 nm for EGFP (PicoQuant PLS 450, 800 ps), 500 nm for EYFP and TagRFP-T (PicoQuant PLS 500, 800 ps), or 600 nm for mCherry (PicoQuant PLS 600, 950 ps). The LEDs were operated at a repetition rate of 1 MHz, and photons were collected over a 50 ns time period. All experiments were conducted at 20 °C. Acquisition of fluorescence decay traces for each experimental condition was repeated 5–10 times.

The decay data were analyzed using the deconvolution software part of the FLS980 software (Edinburgh Instruments, UK), which yielded the value of the fluorescence lifetimes (τ_i_) and their amplitudes (α_i_). The quality of the fitting was evaluated through the value of the reduced χ^2^ (≈1.0–1.5) and visual inspection of residuals and autocorrelation of residuals.

### 2.4. Fluorescence Microscopy for Detection of NO by mTagBFP2-Expressing HeLa Cells

HeLa S3 cells were cultivated in Dulbecco’s modified Eagle’s medium (DMEM) (Sigma-Aldrich) with 10% FCS, penicillin (100 U/mL), streptomycin (100 µg/mL), amphotericin (1.25 µg/mL), 1 g/L glucose, and 4 mM glutamine at 37 °C in a humidified incubator with 5% CO_2_. Forty-eight hours before measurements, the cells were seeded onto 30 mm circular glass slides (Paul Marienfeld GmbH, Lauda-Königshofen, Germany). Twenty-four hours before measurements, the cells were transfected with 1 mL of serum- and antibiotic-free medium containing 1.5 µg of the appropriate plasmid DNA (FSy 1.1-mTagBFP2, Fsy 1.1-mTagBFP2 C26A C114S C222S, pcDNA3.1-C-geNOp [20], and 2.5 mg of TransFast transfection reagent (Promega, Madison, WI, USA)) for 3 h, according to the manufacturer’s guidelines, followed by a medium change and cultivation until measurements were performed. For live-cell measurements, the coverslips containing the cells were inserted into a PC30 perfusion chamber (NGFI, Graz, Austria) and connected to a gravity-based perfusion system (NGFI). For equilibration, a physiological buffer (2 mM CaCl_2_, 135 mM NaCl, 1 mM MgCl_2_, 5 mM KCl, 10 mM HEPES, 10 mM glucose; pH 7.4) was perfused for several minutes, until a stable baseline was observed. Subsequently, the cells were perfused with physiological buffers containing 3 mM sodium nitroprusside (SNP, Sigma-Aldrich). The cells were imaged on an Olympus IX73 inverted microscope equipped with a 40× oil immersion objective (UApo N 340, 40×/1.35 Oil, ∞/0.17/FN22, Olympus, Wien, Austria). Cells were excited at 385 nm using a LED-based light source (LedHUB, OMICRON electronics, Rodgau-Dudenhofen, Germany), and emission was collected at 480 nm (ET480/40 m, Chroma Technology Corp., Bellows Falls, VT, USA) using a Retiga R1 CCD-camera (TELEDYNE QIMAGING, Surrey, BC, Canada).

### 2.5. Sequence Alignment

The sequences of the proteins studied in this work were aligned using CLUSTAL O (1.2.4) [28].

## 3. Results

### 3.1. Effect of NO on mTagBFP2 Fluorescence Emission

Before exposing mTagBFP2 to nitric oxide, protein solutions were extensively flown with pure nitrogen for 40 min in order to remove dissolved oxygen. The NO concentration was then progressively increased by anaerobically adding proper amounts of a concentrated NO solution. The absorption spectrum of mTagBFP2 was essentially unaffected by NO, except for an increase in absorption in the far UV, possibly receiving contribution from decomposition products of the NO donor. An increase in light scattering was also observed.

**Figure 1:**
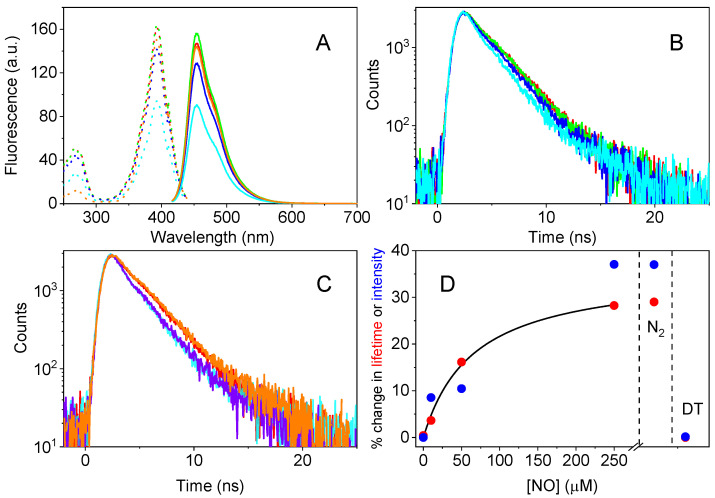
(**A**) Fluorescence excitation (dotted lines) and emission (solid lines) spectra of mTagBFP2 in PBS after deoxygenation with N_2_ (red) and at different NO concentrations: [NO] = 10 µM (green), [NO] = 50 µM (blue), and [NO] = 250 µM (cyan). The sample was then exposed to N_2_ flow for three hours and subsequently reduced with Na dithionite (2 mM), recovering initial properties (orange curves). For emission spectra λ_ex_ = 400 nm and for excitation spectra λ_em_ = 460 nm. (**B**) Time-resolved fluorescence (TCSPC) for mTagBFP2 in PBS. The color code is the same as in panel (A). Excitation for time-resolved fluorescence was at 380 nm, and detection at 460 nm. (**C**) Reversibility of the time-resolved fluorescence signal after exposure of mTagBFP2 to NO. The curves refer to mTagBFP2 in PBS after deoxygenation with N_2_ (red), in the presence of [NO] = 250 µM (cyan), after flowing N_2_ for 1 h (purple), and after reduction with Na dithionite (orange). (**D**) Percent change in an average lifetime (red circles) and fluorescence emission intensity (blue circles). The black solid line is the result of the fit of the lifetime data with a model for binding of NO to the protein. The plot also shows the effect of N_2_ saturation for 1 h (N_2_) and the subsequent reduction with Na dithionite (DT). The experimental values for percent change in fluorescence lifetime and intensity after Na dithionite reduction coincided.

Fluorescence excitation and emission spectra showed a progressive decrease in intensity when NO was added, with no appreciable change in shape (Figure 1A). Consistently, the fluorescence decay became faster at increasing NO concentrations (Figure 1B). At all NO concentrations, the decay was best described by a double exponential relaxation (Table S1), with lifetimes of 2.74 ± 0.28 ns (88%) and 0.62 ± 0.06 ns (12%) in the nitrogen-saturated solution. This corresponded to an average lifetime of <τ> = (2.49 ± 0.04) ns. The average lifetime became (1.78 ± 0.02) ns when the NO concentration was increased to 250 μM.

Figure 1D plots the percent change of the average fluorescence lifetime (red circles) and the fluorescence emission intensity (blue circles) as a function of NO concentration. The trend suggests that a binding reaction occurred, with the steady-state fluorescence data being noisier than those from fluorescence lifetime. At the highest NO concentration, light scattering of the 380 nm excitation beam may be responsible for the artificially higher intensity quenching in comparison with the time-resolved experiment, for which the effect of scattering was expected to be negligible. An estimate of the equilibrium dissociation constant *K*_d_ was obtained with a simple binding model. The black solid line is the result of the best fit with a hyperbolic binding isotherm to a single binding site, with *K*_d_ = (70 ± 10) μM. The saturation change was (36 ± 2)% at high NO concentrations. Fitting parameters are reported in Table 1.

Extensive (3 h) flowing of nitrogen of the NO equilibrated protein solution was unable to recover the spectroscopic properties of the initial nitrogen saturated solution. After the anaerobic addition of 2 mM Na dithionite, the fluorescence excitation, emission spectra, and fluorescence decay became identical to the ones obtained before the reaction with NO (Figure 1C).

Although the binding of NO appears to be fully reversible, accomplishing dissociation of NO from mTagBFP2 requires the use of a reducing agent. The above findings suggest that S-nitrosylation, i.e., the covalent attachment of nitric oxide to the thiol side chain of cysteine (Scheme 1), may occur in mTagBFP2, and this reaction may have been responsible for the observed changes in fluorescence lifetime and intensity. Indeed, preliminary mass spectrometry (MS) on mTagBFP2 exposed to the nitroso donor S-nitrosoglutathione, showed that two out of three Cys residues were nitrosylated (Figure S1). Future work will include a thorough investigation of the sites of nitrosylation by performing an MS characterization of mtagBFP2 and different Cys mutants (and similarly for other FPs such as mTagRFP-T, vide infra) in order to clarify which Cys residues are responsible for the observed fluorescence quenching.

### 3.2. Identification of the Role of Cys Residues

**Figure 2:**
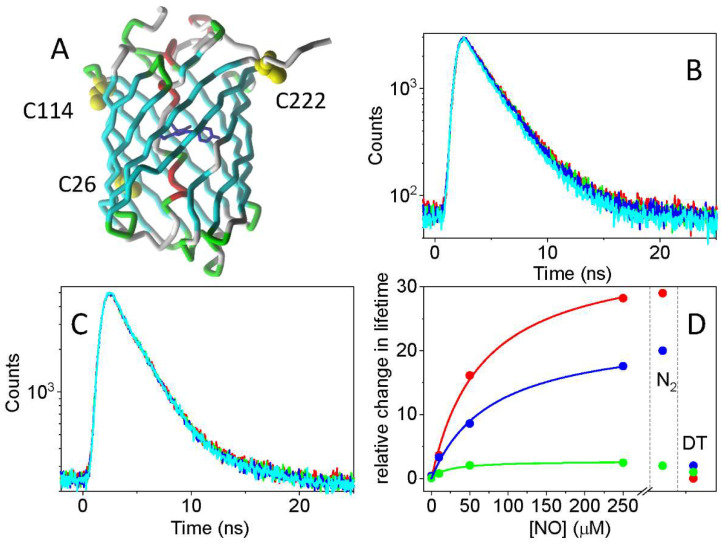
(**A**) Schematic representation of the three-dimensional structure of mTagBFP2, where C26, C114, and C222 are represented as yellow solid spheres, and the chromophore as blue sticks. (**B**) Fluorescence decays of (C26A) mTagBFP2 in PBS after deoxygenation with N_2_ (red) and at different NO concentrations: [NO] = 10 µM (green), [NO] = 50 µM (blue), and [NO] = 250 µM (cyan). (**C**) Fluorescence decays of (C26A C114S C222S) mTagBFP2 in PBS after deoxygenation with N_2_ (red) and at different NO concentrations: [NO] = 10 µM (green), [NO] = 50 µM (blue), and [NO] = 250 µM (cyan). After flowing N_2_ for three hours (no change of fluorescence lifetime) and subsequent reduction with Na dithionite, the initial signals were restored for both (C26A C114S C222S) mTagBFP2 and (C26A) mTagBFP2. (**D**) Percent change in average lifetime for mTagBFP2 (red circles), (C26A) mTagBFP2 (blue circles), and (C26A C114S C222S) mTagBFP2 (green circles). The data after N_2_ equilibration (N_2_) and reduction with Na dithionite (DT) are reported as well. The solid lines are the result of the fits of the lifetime data with a model for binding a small ligand (NO) to the macromolecule.

Three Cys residues were present in mTagBFP2, located at positions 26, 114, and 222 (Figure 2A) [19]. To assess the role of Cys residues in the observed effect, we generated the single-mutant (C26A) mTagBFP2 and the triple-mutant (C26A C114S C222S) mTagBFP2 by site-directed mutagenesis. When C26 was mutated to A, the shape of absorption, fluorescence excitation, and fluorescence emission spectra were not affected (data not shown). The average fluorescence lifetime for N_2_-saturated solutions was not much different from the one observed for the wt protein (2.46 ± 0.04) ns. The fluorescence decay, reported in Figure 2B, showed a weaker change upon increasing NO concentration with an average lifetime of 2.02 ± 0.03 ns at [NO] = 250 μM. Consequently, the observed percent change in average fluorescence lifetime (Figure 2C,D) was smaller than for mTagBFP2 (23 ± 2% at saturation), although the dissociation constant was not much different, *K*_d_ = (80 ± 10) μM. Similar results were obtained from fluorescence emission intensity (data not shown). Flowing mTagBFP2 (C26A) solution containing 250 μM [NO] with N_2_ did not affect fluorescence intensity or lifetime (see Figure 2D). Reduction with Na dithionite recovered initial fluorescence decay properties including average lifetime (see Figure 2D).

When all three cysteines were mutated in (C26A C114S C222S) mTagBFP2, the fluorescence decay curves collected at increasing NO concentrations were barely distinguishable from each other (Figure 2C). Accordingly, the observed change in lifetime was almost negligible (3 ± 1% at 250 μM) (Figure 2D), showing that the NO sensitivity of the fluorescence emission was removed.

These findings were conclusive evidence that S-nitrosylation of Cys residues is the basis of the observed effects on fluorescence lifetime (and intensity). The need for a reducing agent to restore fluorescent properties when NO is removed should not be taken as a limitation in the application of these fluorescent sensors. The redox potential of the cell may be sufficient to reduce nitrosothiols upon removal of NO in the vicinity of mTagBFP2, as will be demonstrated in a later subchapter (mTagBFP2 as a NO sensor in living cells).

### 3.3. Effect of NO on Other Fluorescent Proteins

The above findings suggest that also in other fluorescent proteins, a similar mechanism may lead to changes in fluorescence emission properties in the presence of NO, due to S-nitrosylation with cysteine residues and subsequent quenching of the fluorescence emission. We, therefore, tested some fluorescent proteins, which are prominently used in fluorescence microscopy in cell biology and life science applications, covering different fluorescence emission colors (EGFP, EYFP, TagRFP-T, mCherry), including one devoid of cysteine residues (mCherry).

**Figure 3:**
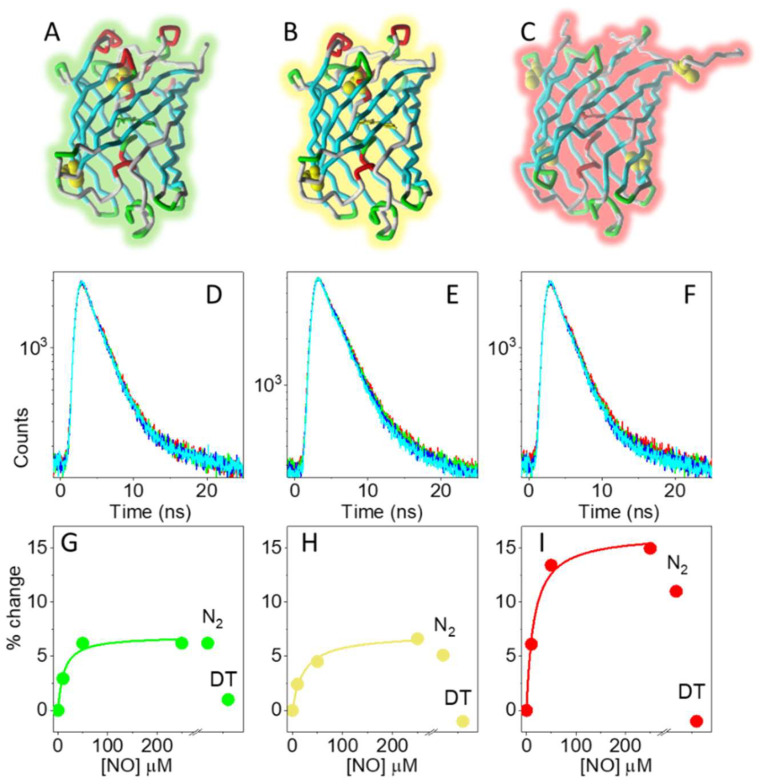
Schematic structures of EGFP (**A**), pdb code 2y0g), EYFP (**B**), pdb code 1YFP), and TagRFP-T (**C**), pdb code 5jva). Cys residues are displayed as yellow CPK, and chromophores are displayed as sticks. Lower panels show the fluorescence decays measured at different concentrations of NO: N_2_ saturated (red), [NO] = 10 µM (green), [NO] = 50 µM (blue), and [NO] = 250 µM (cyan). (**D**) EGFP. (**E**) EYFP. (**F**) TagRFP-T. Percent changes in fluorescence lifetimes for the three proteins as a function of [NO] are plotted in (**G**) EGFP, (**H**) EYFP, and (**I**) TagRFP-T. The plots also show the effect of N_2_ saturation for 1 h (N_2_) and the subsequent reduction with Na dithionite (DT). The solid lines are the result of the fits of the lifetime data with a model for binding a small ligand (NO) to the macromolecule.

Figure 3 compares the three-dimensional structures of EGFP (panel A), EYFP (panel B), and TagRFP-T (panel C). EGFP and EYFP have two Cys residues in identical positions (48 and 70). Not surprisingly, their response to NO binding is quite similar both in terms of maximum percent change in lifetime (7 ± 1% for EGFP and 7 ± 1% for EYFP) and dissociation constant (10 ± 5 μM for EGFP, Figure 3D,G, and 20 ± 6 μM for EYFP, Figure 3E,H). Although small, the effect is perfectly reproducible and is fully reversible when Na dithionite is added.

TagRFP-T has four Cys residues (26, 114, 172, and 222, Figure 3C), and NO binding resulted in a larger (15 ± 1%) lifetime change (Figure 3F,I), with a *K*_d_ = 15 ± 4 μM. Also for this fluorescent protein, the addition of Na dithionite allowed for the recovery of the fluorescence properties observed before the reaction with NO.

In an attempt to further prove the role played by Cys residues, we engineered a Cys-free variant of TagRFP-T. However, the protein would not express correctly, and we were unable to verify the disappearance of the NO sensitivity upon removal of Cys residues. We thus took the red mFruit variant mCherry, a second-generation monomeric red fluorescent protein with improved brightness and photostability, for which no Cys residues are present in the amino acid sequence [29].

**Figure 4:**
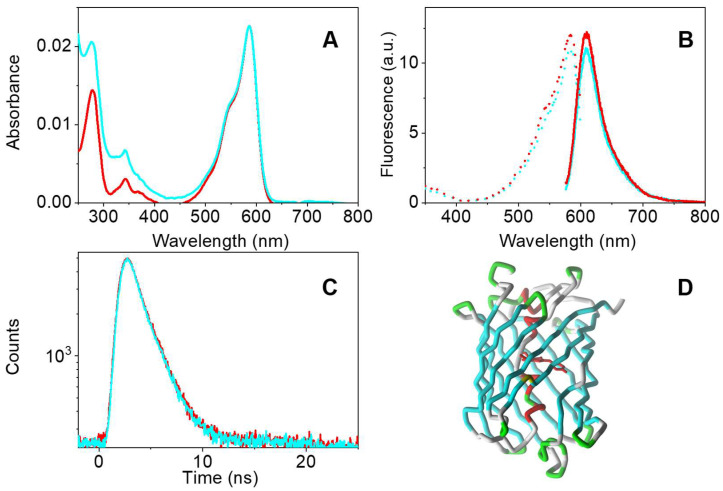
(**A**) Absorption spectra of a mCherry solution equilibrated with N_2_ (red) and in the presence of [NO] = 250 µM (cyan). (**B**) Fluorescence excitation (dotted lines) and emission (solid lines) spectra of a mCherry solution equilibrated with N_2_ (red) and in the presence of [NO] = 250 µM (cyan). (**C**) Fluorescence decays of mCherry solution equilibrated with N_2_ (red) and in the presence of [NO] = 250 µM (cyan). (**D**) Schematic structure of mCherry with the chromophore displayed as red sticks (pdb code 2H5Q).

Figure 4A–C demonstrates that NO did not affect the spectral properties of mCherry. The changes in the absorption spectrum, fluorescence excitation and emission, and fluorescence decay were negligible.

This finding confirms the correlation with the observed NO effect on fluorescence emission for Cys bearing fluorescent proteins.

### 3.4. Effect of pH on NO Sensitivity

The above properties of mTagBFP2 and TagRFP-T may be exploited to monitor intracellular NO production under conditions where high local concentrations are expected such as in lysosomes during the response of lymphocytes to attacks by pathogens [30,31]. In such an environment, the pH is well below neutrality, and it may influence fluorescence emission. We thus repeated the above experiments at pH 5 in order to assess the possible changes in response to NO in acidic environments.

**Figure 5:**
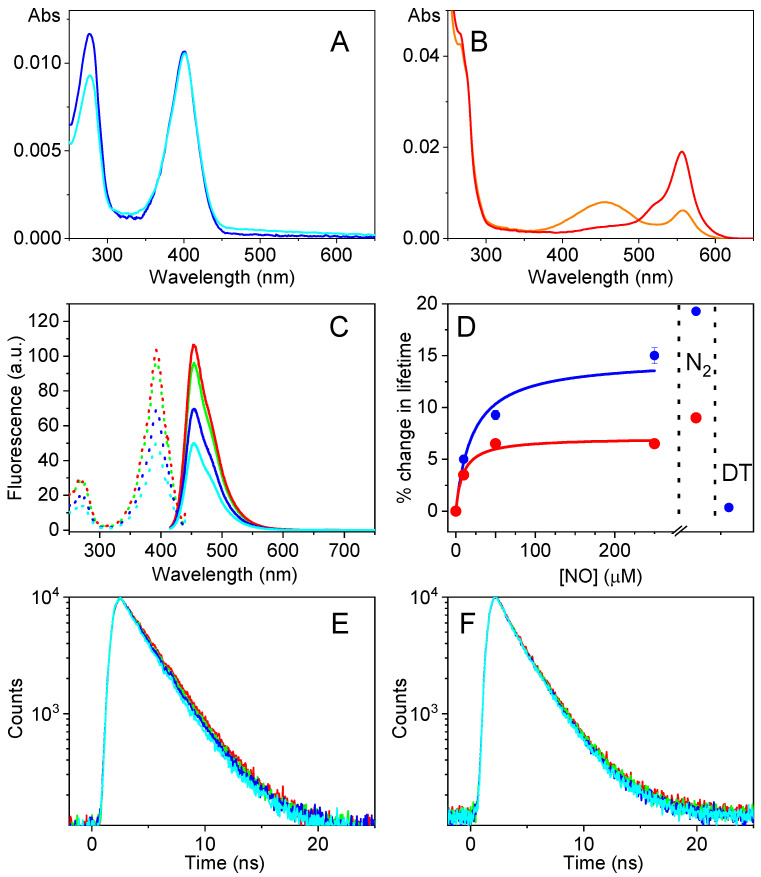
(**A**) Comparison between absorption spectra of mTagBFP2 at pH 7.4 (cyan) and pH 5 (blue). (**B**) Comparison between absorption spectra of TagRFP-T at pH 7.4 (red) and pH 5 (orange). Fluorescence excitation (dotted lines) and emission (solid lines) spectra (**C**) and fluorescence decays (**E**) of mTagBFP2 in acetate buffer at pH = 5 after deoxygenation with N_2_ (red) and at different NO concentrations: [NO] = 10 µM (green), [NO] = 50 µM (blue), and [NO] = 250 µM (cyan). The sample was then exposed to N_2_ flow for three hours (N_2_) and subsequently reduced with Na dithionite (2 mM) to revert NO binding (DT). For emission spectra, λ_ex_ = 400 nm, and for excitation spectra, λ_em_ = 460 nm. Excitation for time-resolved fluorescence was at 380 nm, and detection was at 460 nm. (**F**) The fluorescence decays for TagRFP-T in acetate buffer at pH = 5. The color code was the same as for (**E**). Excitation was at 500 nm, and detection at 585 nm. (**D**) The percent change in average lifetimes for TagRFP-T (red circles) and mTagBFP2 (blue circles). The solid lines are the results of the fits of the lifetime data with a model for binding of NO to the protein.

In the case of mTagBFP2, the average fluorescence lifetime changed from 2.49 ± 0.04 ns at pH 7.4 to 2.80 ± 0.05 ns at pH 5 in N_2_-saturated solutions. The low p*K*_a_ of the chromophore (around 2.7 [18,19]) was consistent with the observed very small spectral changes (see Figure 5A).

For TagRFP-T, with a chromophore p*K*_a_ of 4.6 [19,32], a considerable amount of the protein existed with the chromophore in its neutral form at pH = 5 with an increase in 450 nm absorption and a concomitant decrease in the 550 nm absorption band (Figure 5B). For this variant, the average fluorescence lifetime changed from 1.93 ± 0.04 ns at pH 7.4 to 2.00 ± 0.03 ns at pH 5.

The effect of NO on fluorescence emission intensity (Figure 5C) and lifetime (Figure 5E,F) became smaller but was still clearly detectable. For mTagBFP2, we observed a maximum change of 16 ± 1% and a *K*_d_ = 35 ± 10 μM, whereas for TagRFP-T, we obtained a maximum change of 7 ± 1% and a *K*_d_ = 10 ± 4 μM (Figure 5D).

### 3.5. mTagBFP2 as a NO Sensor in Living Cells

NO is an important signaling molecule and biomarker [33,34], but also a cell toxic species [35,36]. It is therefore desirable to observe NO concentrations in living cells and biological tissue by light and more specifically by fluorescence microscopy. However, the number of fluorescent NO sensors available is limited, and their performance is not optimal [21,37]. Here, for the first time we described NO sensitivity of the fluorescence of mTagBFP2 (and other FPs), in this context an interesting finding, potentially being the starting point for the development of a new class of fluorescent NO sensors. 

**Figure 6:**
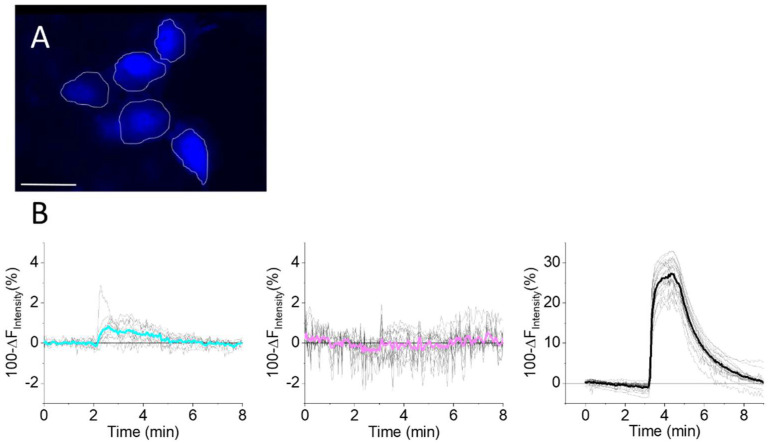
(**A**) HeLa cells expressing mTagBFP2. Scale bar represents 20 µm. (**B**) Fluorescence intensity over time of HeLa cells expressing mTagBFP2 (left), mTagBFP2 C26A C114S C222S (middle), and C-geNOp (right) in response to 3 mM SNP perfused at around 2.5 min, causing NO release. Note that the sign of fluorescence change is inverted due to plotting 100 - ΔF_Intensity_ (%).

To strengthen this idea and stimulate further research in this area, we performed NO-sensing fluorescence microscopy experiments with mTagBFP2 expressed in HeLa cells (Figure 6A), a frequently used human cancer cell line. While constantly exciting and observing the fluorescence intensity of mTagBFP2, the cellular concentration of NO was increased by the transient influx (1 min) of a NO-liberating SNP in the extracellular solution at time point of 2.5 min (3 mM SNP concentration). The addition of SNP significantly quenched the fluorescence of HeLa cells (Figure 6B, left image), indicating that intracellular NO affects the mTagBFP2 fluorescence. Note that the fluorescence was plotted as relative change and with an inversed sign. In this way, a positive signal indicates an increased NO concentration. After removal of the NO-releasing substance, the fluorescence of mTagBFP2 returned to the basal level, pointing to a reversal effect of NO on the FP. The left panel in Figure 6B shows the complete time course of mTagBFP2 fluorescence change for 10 cells (in cyan, the average trace). This experiment indicated that mTagBFP2 can detect NO in cells in a reversible manner. In addition, we also performed two control experiments. Most importantly, the cys-free mutant mTagBFP2 C26A C114S C222S (15 cells) showed no fluorescence change upon NO release at around 2.5 min (Figure 6B, middle image; in magenta, the average trace), while the established NO sensor C-geNOp showed a robust signal at the same time point when NO was released from SNP (Figure 6B, right panel; in black, the average trace over 20 cells). The much larger signal change observed in the case of C-geNOp was mostly due to the higher affinity for NO of this sensor. Further physiological measurements, as well as improvement of the sensing properties by mutations in mTagBFP2 and other FPs, are planned for the future.

## 4. Discussion

The introduction of GFP and its many variants as a tool has revolutionized fluorescence-microscopy-based studies in cell biology. Besides the possibility to endow proteins with different colors and observe their localizations in real-time, GFP-like fluorescent proteins are the basic building block for genetically encoded biosensors. Genetically encoded fluorescent sensors usually include some sensitive protein domains that determine a specific response to a particular analyte or activity [14,38]. Since the appearance of the first ones (for [Ca^2+^] and pH), several more have been developed to monitor a wide variety of cellular parameters, including ion concentrations and redox power, but also the activity of kinases or phosphatases.

In this work, we describe a new, not yet described posttranslational modification of GFPs, namely, S-nitrosylation of cysteine residues (see Scheme 1), which modulates the fluorescence brightness of FPs. The reaction occurs at micromolar NO concentrations and is therefore a rare phenomenon under normal physiological conditions, where NO concentration is in the nanomolar range [39]. In inflammation processes, however, myeloid cells such as macrophages generate high [NO], which is used for the killing of pathogens [40,41].

**Figure 7:**
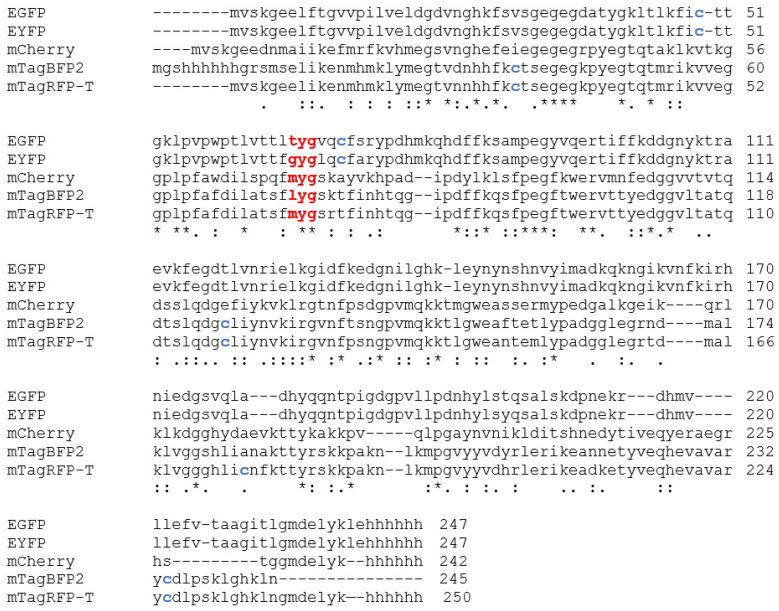
Sequence alignment for the proteins studied in this work. Proteins have been aligned using Clustal 2.1 multiple alignment tool. Stars (*) indicate identities and dots indicate a higher (:) and a lower (.) degree of similarity. Cys residues are highlighted in blue. Residues in red are involved in chromophore formation and were used for sequence alignments (note the hexa-histidine tag used for purification, which is at the C-terminus, except for mTagBFP2, where it is at the N-terminus).

We first established the NO-induced fluorescence decrease in mTagBFP2. The chromophore is not directly affected since the shape of absorption and fluorescence spectra are unchanged. Instead, a putative fluorescence quenching process caused by NO, which leads to a faster deactivation of the emissive state, explains the observed fluorescence intensity decrease and the concomitant fluorescence lifetime shortening. We then speculated that NO would not directly interact with the chromophore but may react with one or all of the three cysteines of mTagBFP2 in a reaction called S-nitrosylation (Scheme 1). The reduction and disappearance of the NO-induced fluorescence quenching, respectively, in two mTagBFP2 mutants with one or all cysteines replaced by alanine or serine proved that the cysteines are indeed a requirement for the fluorescence quenching and indicated that no particular cysteine in mTagBFP2 is responsible for the NO effect. Instead, the process seems to be a general phenomenon for GFP-like FPs, since we found similar fluorescence quenching in other cysteine-containing FPs (EGFP, EYFP, mTagRFP-T), while the fluorescence quenching was absent in another, cysteine-free fluorescent protein (mCherry). Figure 7 provides an overview of the cysteine positions in the five fluorescent proteins studied here (with mCherry having no cysteine). They were not concentrated near the chromophore and were different from the FPs originating from different wild-type FPs (EGFP/EYFP and mTagBFP2/mTagRFP-T).

**Figure 8:**
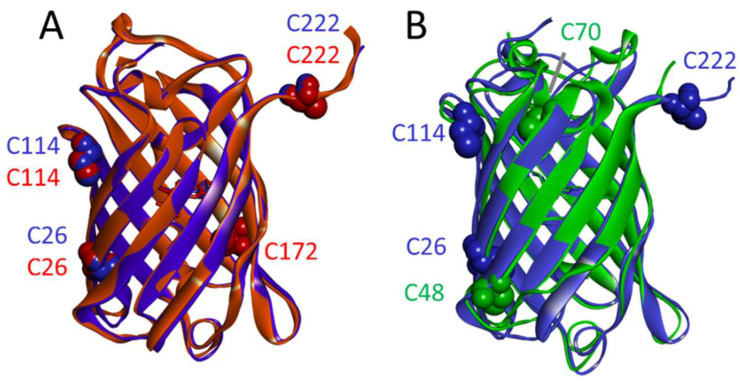
(**A**) Superposition between the structure of mTagBFP2 (blue solid ribbon, PDB 3m24) and mTagRFP-T (red solid ribbon, PDB 5jva). Cys residues are displayed as a space-filling model. (**B**) Superposition between the structure of mTagBFP2 (blue solid ribbon, PDB 3m24) and EGFP (green solid ribbon, PDB 2y0g).

The topological position of the common Cys residues in mTagBFP2 and in mTagRFP-T sequences was essentially identical, as is evident from Figure 8A, where the two structures are superimposed. The only major difference was the additional Cys residue (C172) in mTagRFP-T. This was not the case for the Cys residues in EGFP (and EYFP), whose position was remarkably different from those present in mTagBFP2, as shown in Figure 8B.

To summarize, the position of the Cys residues in mTagBFP2 and in the other fluorescent proteins examined in this work was not consistent with a direct interaction of NO with the chromophore that would explain the effect on fluorescence emission. We rather speculate that the reaction of NO with the Cys side chain affects protein dynamics in a way in which non-radiative relaxation is enhanced, resulting in lower excited state lifetimes.

The exact mechanism of the observed NO sensitivity is admittedly yet to be understood. Nevertheless, we want to present a tentative explanation. It is well established that the rigidity of the β-barrel structure of fluorescent proteins shields the chromophore from the solvent and quenchers. In this way, a favorable environment for fluorescence emission is provided [42,43]. Disruption of barrel integrity leads in general to enhanced contact of solvent molecules to the chromophore, which results in enhanced fluorescence quenching. In addition, a less rigid barrel structure will also offer higher mobility of the enclosed chromophore, which could manifest in more efficient internal conversion as well as increased torsional freedom and photoisomerization of the chromophore. Both processes compete with the radiative deactivation channel and reduce the fluorescence brightness. A well-investigated example is the introduction of an aperture in the barrel, as in the H148G GFP mutant, which strongly affects the protonation state [42,44] but also the oxygen accessibility [43,45] of the chromophore. We propose that the enlargement of the cysteine side chains upon S-nitrosylation (Scheme 1) leads to decreasing rigidity of the β-barrel and a subsequently increased fluorescence quenching. The different position of Cys residues (EGFP/EYFP vs. mTagBFP2/mTagRFP-T) or the presence of additional Cys residues (mTagBFP2 vs. mTagRFP-T) may affect the rigidity of the β-barrel upon nitrosylation, and hence fluorescence intensity and lifetime, to a different extent. The specific neighborhood of Cys residues is likely at the basis of the observed different NO affinity. We expect that molecular dynamics simulations may in the future help understanding the role of changes in β-barrel dynamics upon Cys S-nitrosylation and may provide hints to develop mutants with improved sensitivity. The admittedly low, although highly reproducible, changes in fluorescence lifetime/intensity could thus be improved by suitably engineered Cys residues at positions with a larger impact on protein dynamics. 

We also examined whether the NO-induced fluorescence quenching would be present at acidic pH (pH 5), which is important if one would like to detect NO concentrations in, e.g., phagosomes (see below). We observed smaller but still well detectable fluorescence quenching in comparison to those observed in neutral solutions. This finding may be related to a decreased reactivity of some of the Cys residues at acidic pH [46]. Another explanation would be a lesser effect of the bulkier cysteine side chains after S-nitrosylation on the rigidity of the FP’s β-barrel, simply because the β-barrel might be already less rigid due to protonation of other amino acid side chains at low pH. It is important that fluorescence quenching due to the presence of NO still occurs.

Another important aspect is the stability of the bond formed between NO and the sulfur atom of the cysteine side chain. While in bare PBS, the smaller fluorescence lifetime and the decreased fluorescence intensity were present for many minutes, these fluorescence parameters could be reversed to original values by the addition of a reducing agent. When mTagBFP2 was investigated in living cells, however, the fluorescence parameters returned to normal on a time scale of seconds, which can be explained by the redox power of the cell’s cytoplasm. This is an important finding for using S-nitrosylation of cysteines in GFP-like fluorescent proteins as a new type of NO biosensor since it shows that the biosensor’s signal is reversible and can detect subsequent periods of high and low NO concentrations.

We excluded the fact that specific interactions between mTagBFP2 and other cell constituents might be the cause of the observed quenching. mTagBFP2 is a water-soluble protein with no known specific interactions, and its distribution in all performed experiments with mammalian cells was quite uniform, with no indication of accumulation in specific areas (Figure S2A,C). This was the case also for mTagBFP2-overexpressing *E. coli* cells (Figure S3). Moreover, the average lifetime of mTagBFP2 fluorescence emission was identical for the purified recombinant protein in PBS buffer and inside *E. coli* or HeLa cells (Figures S2B,D and S4).

As a further control, we also performed an experiment in the presence of 100 µM bovine serum albumin, where no changes in absorption, fluorescence excitation/emission spectra, or fluorescence lifetime of mTagBFP2 (Figures S5 and S6) was observed in comparison with the lifetime observed in PBS buffer (Table 1).

Cysteines in GFP-like fluorescent proteins have been previously exploited for sensing different cellular properties, namely, the intracellular and intra-organelle redox potential. Cysteines at positions favorable for disulfide bridge formation were engineered to introduce redox potential sensitivity in the fluorescence emission by a fluorescent protein termed roGFP [13,47]. The crystal structure demonstrated the reversible formation of the disulfide bridge for roGFPs and suggested that localized geometric strain caused by disulfide formation propagates toward the chromophore region, which presumably accounts for the redox-dependent shifts in the excitation spectrum. roGFPs were reported to have two fluorescence excitation maxima at about 400 and 490 nm and to display rapid and reversible ratiometric changes in fluorescence in response to changes in ambient redox potential [13]. The position of His148 and Ser205, which contact the chromophore, was suggested to be affected by disulfide bond formation/rupture between the introduced cysteine residues 147 and 204. Thus, changes in the chromophore environment were suggested to perturb the equilibrium between the neutral and anionic forms of the chromophore and result in the observed response to redox changes.

The NO sensitivity of the fluorescence of GFP-like fluorescent proteins, which we have demonstrated here for the first time, is not only interesting as such but may be the starting point for the development of NO biosensors on the basis of cysteine-rich fluorescent proteins. The existing fluorescent NO probes are far from optimal. The only genetically encodable biosensor requires special treatment for cofactor loading. The replacement of these by simpler biosensors on the basis of the fluorescence quenching due to S-nitrosylation of cysteines in fluorescent proteins—natural or engineered—is a future goal of this line of research. The fluorescent proteins examined in this work represent one of those rare cases when the fluorescent protein itself is sensitive to the analyte. As such, it is extremely appealing for the development of genetically encoded NO sensors. The small size of the proteins can be easily engineered in a fusion protein endowed with targeting capability, thus allowing real-time, live-cell monitoring of NO in a variety of organelles such as, e.g., lysosomes, autophagosomes, or phagosomes.

A major problem in conventional fluorescence microscopy, which is based on registering fluorescence intensities, in quantitative cellular measurements, is the impossibility of controlling or determining the number of fluorophores inside a cell. Since fluorescence intensity is directly proportional to the number of fluorophores, it requires additional calibration measurements to obtain quantitative results. This is true for both genetically encoded fluorescent proteins and fluorescent organic molecules enriched in cells, where both expression and enrichment levels may differ significantly from cell to cell. Fluorescence lifetime on the other hand is not dependent on the number of fluorophores, and therefore, fluorescence lifetime imaging is often the method of choice when accurate absolute measurements are required in fluorescence microscopy applications [48,49,50]. mTagBFP2 already owns a fluorescence lifetime contrast sufficient for FLIM measurements to determine NO concentration. The intended future improvements in fluorescence quenching strength as well as affinity may result in the development of an easy-to-use, genetically encoded fluorescent NO sensor for quantitative NO determination in living cells via FLIM.

## 5. Conclusions

We report here the discovery of a posttranslational modification (S-nitrosylation of Cys residues) in GFP-like fluorescent proteins and in particular in mTagBFP2, which leads to a reduction of fluorescence intensity and lifetime. On the basis of this posttranslational modification, we propose the constructions of NO biosensors. While the performance of the proposed sensors is yet to be optimized and their full potential yet to be explored, mTagBFP2 and the other variants studied in this work have important advantages over previously proposed genetically encoded fluorescent NO sensors. The small protein size and the dynamic range of the sensitivity are the most relevant points of strength of the sensor. The NO sensitivity is linked to the presence of Cys residues and is fully reversible under the cellular redox potential conditions. Given the low photobleaching of mTagBFP2, this sensor allows for the monitoring of intracellular NO dynamics for extended times. Due to the principle of the method, the affinities of the proteins for NO enable sensing of this analyte in the µM concentration range, which qualifies the sensor as a potential tool for investigations on macrophage response to microbial infections [51]. An additional point of strength of our findings is that the fluorescent proteins of different hues we tested may expand the palette of fluorescent NO sensors to cover the whole visible spectrum. Finally, mTagBFP2 (and the other variants) appear to be promising candidates as genetically encoded NO sensors for fluorescence lifetime imaging applications.

## Figures and Tables

**Table 1:** Dissociation constants and maximum percent change for NO binding to investigated fluorescent proteins, derived from lifetime data. The average fluorescence lifetime in the absence of NO is reported for reference.

Protein	*K*_d_ (μM)	Max % Change	<τ> (ns)
**mTagBFP2**			
pH = 7.4	70 ± 10	36 ± 2	2.49 ± 0.04
pH = 7.4 + BSA 100 µM	--	--	2.50 ± 0.05
pH = 5	35 ± 10	16 ± 1	2.80 ± 0.05
C26A	80 ± 10	23 ± 2	2.46 ± 0.04
C26A C114S C222S	--	3 ± 1	2.35 ± 0.02
**EGFP**	10 ± 5	7 ± 1	3.35 ± 0.04
**EYFP**	20 ± 6	7 ± 1	2.76 ± 0.05
**TagRFP-T**			
pH = 7.4	15 ± 4	16 ± 1	1.93 ± 0.04
pH = 5	10 ± 4	7 ± 1	2.00 ± 0.03
**mCherry**	--	0	0.97 ± 0.02

## Data Availability

Data available on request due to restrictions e.g., privacy or ethical. The data presented in this study are available on request from the corresponding authors.

## Supplementary Materials

The following supporting information can be downloaded at: https://www.mdpi.com/article/10.3390/antiox11112229/s1, Figure S1: Deconvolution of mass spectrum; Figure S2: Two-photon excitation Fluorescence Lifetime Imaging (2PM-FLIM) of HeLa cells transfected with mTagBFP2 and mTagBFP2 C26A C114S C222S and the corresponding fluorescence decays; Figure S3: Bright field and corresponding fluorescence images of *E. coli* (BL21) expressing mTagBFP2; Figure S4: Fluorescence emission and excitation spectra of an *E. coli* (BL21) suspension overexpressing mTagBFP2; Figure S5: Absorption and fluorescence emission spectra of mTagBFP2 in the presence of BSA; Figure S6: Fluorescence decay of mTagBFP2 in the presence of BSA; Table S1: Results of the deconvolution analysis (amplitudes α_i_, lifetimes τ_i_) for the time resolved fluorescence emission of mTagBFP2 at pH 7.4 using a double exponential decay model. Refs [52,53] are cited in the Supplementary Materials File.
