# Supplementary material for: Nitric Oxide Sensing by a Blue Fluorescent Protein
Source: Antioxidants (Basel). 2022 Nov 11;11(11):2229. doi: 10.3390/antiox11112229 (PMC9686608; doi:10.3390/antiox11112229)
Supplement: Supplementary file 1 [file antioxidants-11-02229-s001.zip › antioxidants-2008564-supplementary.pdf]

1. Time Resolved Fluorescence Analysis

The decay data were analyzed using the deconvolution software part of the FLS980 software (Edinburgh Instruments, UK), which yields the value of the fluorescence lifetimes (τ_i) and their amplitudes (α_i). From τ_i and α_i we determined the average lifetime for each decay as: $\langle\tau\rangle = (\alpha_1\tau_1 + \alpha_2\tau_2)/(\alpha_1 + \alpha_2)$. For each experimental condition at least 6 independent decays were collected and analyzed with a double exponential decay model. The fitting parameters averaged over the independent acquisitions are reported in Table S1 for the case of mTagBFP2, for the different experimental conditions we investigated.

Table S1. Results of the deconvolution analysis (amplitudes α_i, lifetimes τ_i) for the time resolved fluorescence emission of mTagBFP2 at pH 7.4 using a double exponential decay model.

mTagBFP2	τ_1 (ns)	α_1 (%)	τ_2 (ns)	α_2 (%)	$\langle\tau\rangle$ (ns)
Deoxy	2.74±0.28	88±3	0.62±0.06	12±3	2.49±0.04
[NO]=10μM	2.70±0.04	85±2	0.64±0.12	15±2	2.39±0.03
[NO]=50μM	2.56±0.07	75±2	0.64±0.07	25±2	2.08±0.02
[NO]=250μM	2.50±0.04	56±4	0.86±0.09	44±4	1.78±0.02
N_2 flow, 3 hrs	2.61±0.04	50±2	0.94±0.07	50±2	1.78±0.03
2 mM Na dithionite	2.85±0.04	82±3	1.07±0.28	18±3	2.53±0.04

2. Mass Spectrometry of S-Nitrosylated Proteins

We have generated S-nitrosylation of mTagBFP2 through incubation with the nitroso donor S-nitrosoglutathione (GSNO) (500 μM final concentration from a DMSO-solubilized stock solution) and carried out a Mass Spectrometry (MS) experiment on the undigested protein using an LTQ Orbitrap (Thermo Fisher Scientific) mass spectrometer (+GSNO in figure S1). The control sample (-GSNO in Figure S1) underwent the same procedure except for GSNO incubation. After 1 h incubation, excess GSNO was eliminated using a SecurityGuard Cartridge Widepore C18 4x2 mm (Phenomenex) column in a DIONEX ULTIMATE3000 (ThermoScientifics) HPLC system. A peak at 27512 Da was detected in the MS of the untreated protein (vs 27639.32 Da theoretical MW, Figure S1, top panel). The 127 Da difference with respect to the primary sequence is consistent with the removal of the N-terminal methionine and with the fluorophore formation. A secondary peak at 26788 is consistent with the partial proteolysis of the affinity tag. Upon incubation with GSNO, both peaks shifted at + 58 Da (Figure S1, bottom panel), consistently with an S-nitrosylation of two residues (with a nitroso group corresponding to +29 Da). Future work on mTagBFP2 mutants (and similarly for other FPs like mTagRFP-T) will clarify which cysteines are responsible for the observed fluorescence quenching.

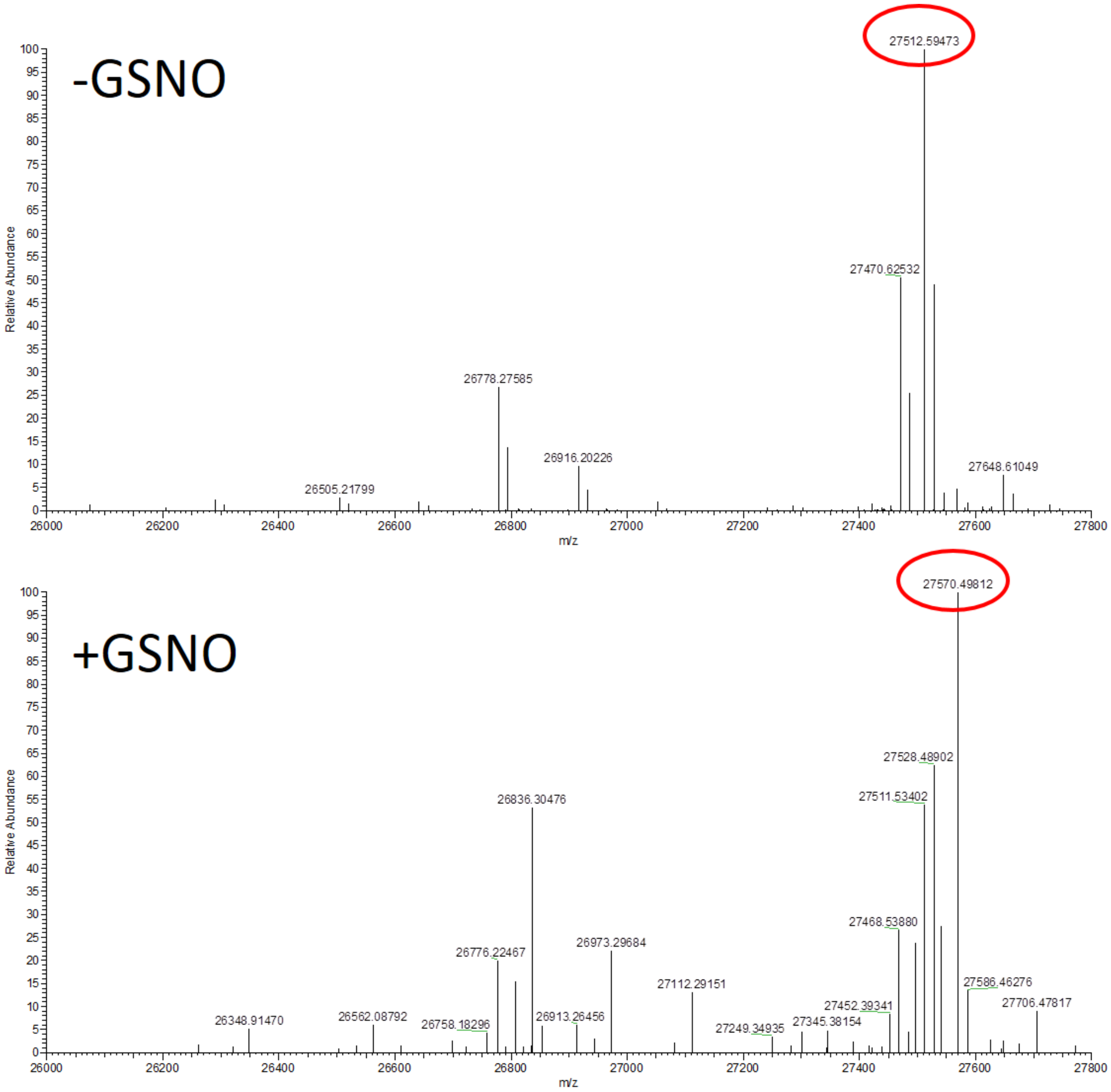

Figure S1. Deconvolution of mass spectrum.

3. Evaluation of Possible Specific Interactions

mTagBFP2 is a water soluble protein with no known specific interactions with other cell constituents. The cellular distribution of mTagBFP2 and C26A C114S C222S mTagBFP2 in all performed experiments with mammalian cells was quite uniform, with no indication of accumulation in specific areas (Figure S2). Moreover, the average fluorescence lifetime of mTagBFP2 and C26A C114S C222S mTagBFP2 was very similar for the purified recombinant proteins in PBS buffer and inside mammalian cells, indicating the absence of quenching by components of nucleoplasm, cytoplasm or other cellular compartments.

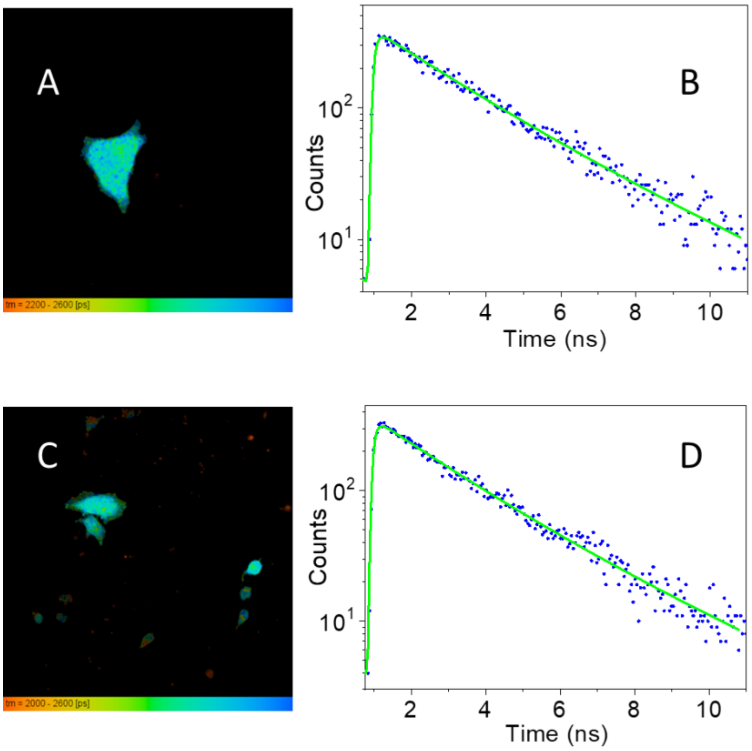

Figure S2. Two-photon excitation Fluorescence Lifetime Imaging (2PM-FLIM) of HeLa cells transfected with mTagBFP2 (A) and mTagBFP2 C26A C114S C222S (C) and the corresponding fluorescence decays (B, D). λ_{ex} = 800 nm. Average lifetimes over the cells were (2.42±0.08) ns (B) and (2.38±0.05) ns (D).

We have also investigated the fluorescence emission from mTagBFP2-overexpressing *E. coli* (BL21). Fluorescence imaging using TIRF geometry (Figure S3) afforded homogenous intensity within the bacteria.

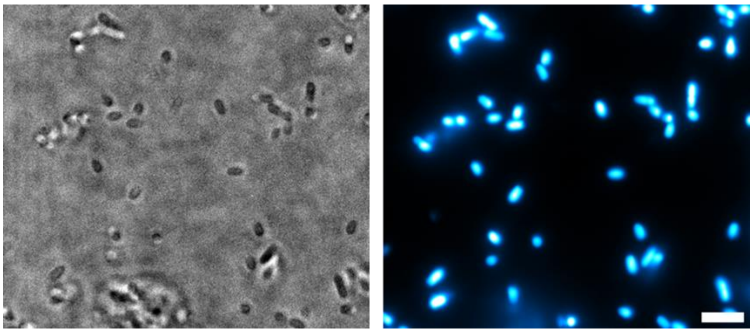

Figure S3. Bright field (left) and corresponding fluorescence (right) images of *E. coli* (BL21) expressing mTagBFP2 (cyan). Scale bar = 5 μm. Excitation wavelength = 405 nm. Images collected with a widefield epifluorescence microscope (ONI nanoimager) in TIRF geometry.

The fluorescence emission of a suspension of *E. coli* (BL21) expressing mTagBFP2 in PBS afforded a lifetime of 2.42±0.05 ns (Figure S4).

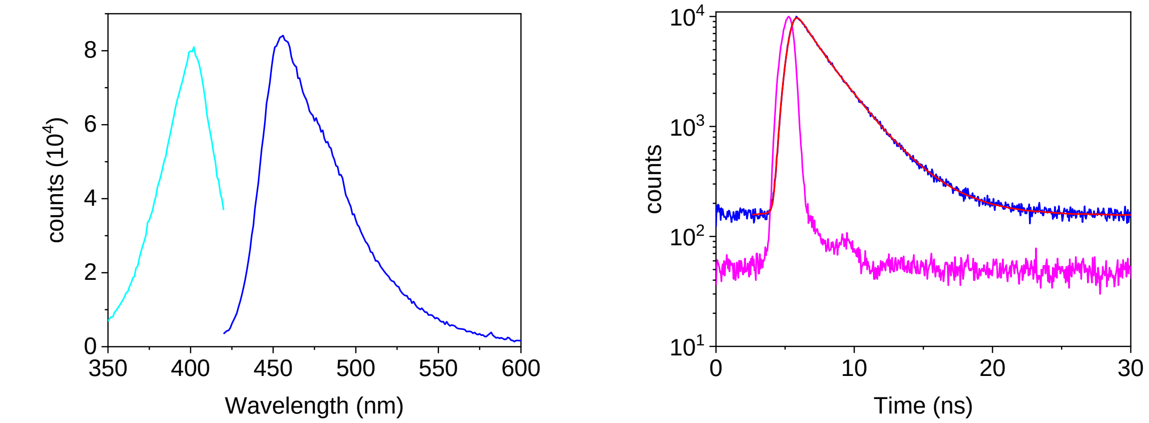

Figure S4. Left. Fluorescence emission (blue) and excitation (cyan) spectra of an *E. coli* (BL21) suspension overexpressing mTagBFP2. Right. Fluorescence decay (TCSPC) of an *E. coli* (BL21) suspension overexpressing mTagBFP2 in PBS (blue, average lifetime 2.42±0.05 ns). The magenta curve shows the IRF. Excitation at 380 nm.

As a further control, fluorescence emission by mTagBFP2 was studied in the presence of BSA at a concentration of 100 μM. No significant changes were observed in the absorption or in the fluorescence excitation and emission spectra of mTagBFP2 upon addition of BSA (Figure S5).

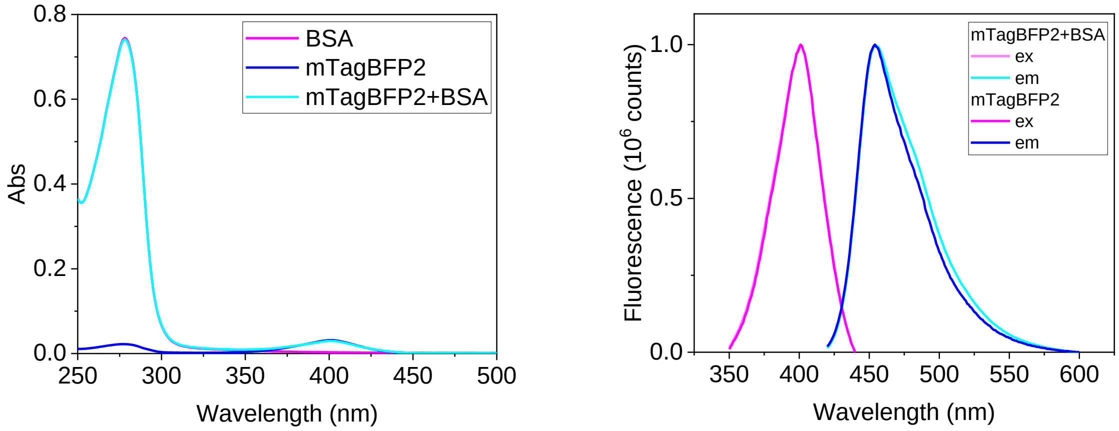

Figure S5. Left. Absorption spectra of BSA (magenta) and mTagBFP2 (blue). The cyan curve represents the absorption spectrum of mTagBFP2 in the presence of 100 μM BSA. Right. Fluorescence emission and excitation spectra of mTagBFP2 in PBS and in the presence of 100 μM BSA. Color code is reported in the panel legend.

Similarly, as can be visually appreciated in Figure S6, no significant changes were observed in the fluorescence decay of mTagBFP2 upon addition of BSA.

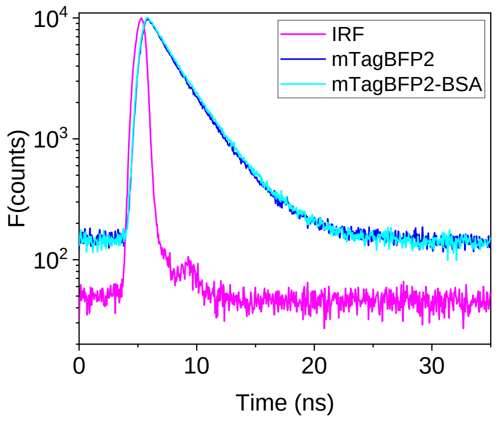

Figure S6. Fluorescence decay (TCSPC) of mTagBFP2 in PBS (blue, average lifetime 2.49±0.04 ns) and in the presence of 100 μM BSA (cyan, lifetime 2.50±0.05 ns). The magenta curve shows the IRF. Excitation at 380 nm.

4. Fluorescence Lifetime Imaging with Two-Photon Excitation, Laser-Scanning Microscopy

HeLa cells expressing mTagBFP2 and mTagBFP2 C26A C114S C222S, respectively, were visualized with a setup described in [1] using an upright fluorescence microscope (A1 MP; Nikon Instruments Europe, Amsterdam, The Netherlands) and observed through a 25x water immersion objective (NA = 1.1; Nikon) at room temperature. Fluorescence was excited with 100-fs light pulses (λ_{exc} = 800 nm) by two-photon excitation. Excitation light pulses were generated at a frequency of 80 MHz by a mode-locked Titan-Sapphire laser (MaiTai DeepSee; output power 2.9 W at 800 nm; Newport (Spectra Physics, Irvine, CA, USA)). The laser light was directed through the lens onto the HeLa cells with reduced power (~8 mW) and scanned over the sample. Fluorescence was recorded by a GaAsP hybrid photodetector (HPM-100-40; Becker & Hickl, Berlin, Germany) using a broad bandpass filter for the whole visible light blocking the intense near-infrared excitation light (bandpass 400–650 nm; Omega Optical, Brattleboro, VT, United States)). Fluorescence lifetime imaging was performed using electronics for time-correlated single photon counting (Simple-Tau 152; Becker&Hickl) and acquisition software (SPCM 9.55; Becker & Hickl) as described before [2]. Lifetime images were analyzed using SPCImage 4.8 (Becker & Hickl) by fitting a bi-exponential model equation to the fluorescence decay in every pixel of the image. The bi-exponential fitting model was necessary and at the same time sufficient to describe fluorescence decays of mTagBFP2 and mTagBFP2 C26A C114S C222S in HeLa cells, as it is the case for the purified proteins in aqueous solution. The program uses an iterative reconvolution of the exponential function with an instrument response function and a least square algorithm for determining parameters for a satisfactory fit.

5. Fluorescence Imaging on Bacteria

Fluorescence images on bacteria were collected with an ONI Nanoimager-S Mark III using a 100X, 1.45 NA oil immersion objective from Olympus. Fluorescence was recorded in the blue-green (498-551 nm) channel of the split sCMOS camera. Fluorescence excitation was obtained with a 405 nm laser (power 22mW). Images were collected over a 50 µm x 80 µm field of view in TIRF geometry.

BL21 *E.coli* bacteria expressing BFP were grown overnight, then washed three times using PBS. For TIRF imaging, bacterial suspensions (approximately 40uL)) were placed in a chamber assembled from a glass slide and a coverslip

(24 x 24 mm, thickness 0.15mm) separated by a double-sided tape. Bacteria were incubated in the chamber for 30 min, and then washed with a large excess of PBS to remove non-adhering cells and the chamber was finally sealed to avoid evaporation. In order to promote bacterial adhesion, clean coverslips were exposed to a poly-L-lysine 0.1mg/mL solution for 30min then dried using nitrogen flow prior to incubation.

References

1. Stölting, G.; de Oliveira, R. C.; Guzman, R. E.; Miranda-Laferte, E.; Conrad, R.; Jordan, N.; Schmidt, S.; Hendriks, J.; Gensch, T.; Hidalgo, P., Direct interaction of CaVβ with actin up-regulates L-type calcium currents in HL-1 cardiomyocytes. *The Journal of biological chemistry* **2015,** *290*, (8), 4561-4572.
2. Kaneko, H.; Putzier, I.; Frings, S.; Kaupp, U. B.; Gensch, T., Chloride accumulation in mammalian olfactory sensory neurons. *The Journal of neuroscience : the official journal of the Society for Neuroscience* **2004,** *24*, (36), 7931-8.
